# ﻿Description of a new subgenus for *Himalaeabatanga* and its new sister species from Xizang Autonomous Region, China (Lepidoptera, Noctuidae, Amphipyrinae, Psaphidini)

**DOI:** 10.3897/zookeys.1244.154162

**Published:** 2025-07-11

**Authors:** Enyong Chen, Alexey M. Prozorov, Aidas Saldaitis, Roman V. Yakovlev, Günter C. Müller, Yonghong Zhou

**Affiliations:** 1 Key Laboratory of Biodiversity and Environment on the Qinghai-Tibetan Plateau, Ministry of Education, School of Ecology and Environment, Xizang University, Lhasa 850000, China; 2 Tibetan Yani Wetland Ecosystem National Observation and Research Station, Xizang University, Lhasa 850000, China; 3 Altai State University, pr. Lenina 61, 656049 Barnaul, Russia; 4 Nature Research Centre, Akademijos str. 2, 08412 Vilnius-21, Lithuania; 5 Tomsk State University, Lenin ave. 36, 634050 Tomsk, Russia; 6 Western Caspian University, 31 Istiglaliyyat St., Baku, Azerbaijan; 7 University of Sciences, Techniques and Technology of Bamako, BP 1805 Bamako, Mali; 8 Kuvin Center for the Study of Infectious and Tropical Diseases, Hadassah Medical School, The Hebrew University, Kalman Ya’akov Man St., 91120 Jerusalem, Israel

**Keywords:** Northeast Himalayan subalpine conifer forests ecoregion, Southeast Tibet shrublands and meadows ecoregion, systematics, taxonomy, Yarlung Zanbo arid steppe ecoregion

## Abstract

A new subgenus, *Macrohimalaea***subgen. nov.**, in the genus *Himalaea* Hreblay & L. Ronkay, 1998, is established and described for Himalaea (Macrohimalaea) batanga Saldaitis, Benedek & Volynkin, 2022 and for a new species, Himalaea (Macrohimalaea) silvana**sp. nov.** This new species is the type of the new subgenus. The new subgenus is distinct from the nominotypical one in having antennal pectination and its forewing shape and male genitalia. All four known adult specimens of the genus and their genitalia are illustrated and compared. Their collection localities are mapped.

## ﻿Introduction

The Qinghai-Xizang Plateau is a 2.5 million km^2^ area with an average altitude of 4000 m. The plateau was possibly elevated after the collision of the Indian and Asian continents about 50 My ago (Searle 2005), or more likely it is a result of much more complex processes originating during the Mesozoic Era (Spicer et al. 2020). The area includes three orogenic hotspots: the mountains of Central Asia, the Himalayas, and the mountains of Southwest China (Hengduanshan) (Mittermeier et al. 2011; Favre et al. 2014 also included part of Indo-Burma). The plateau and consists of more than 20 ecoregions (Olson et al. 2001). The genus *Himalaea* Hreblay & L. Ronkay, 1998, one of the region’s gems, belongs to the subtribe Psaphidina within the tribe Psaphidini of the subfamily Amphipyrinae (Wagner et al. 2008; Lafontaine and Schmidt 2010; Ronkay et al. 2011; Zahiri et al. 2011; Keegan et al. 2019, 2021; Rajaei et al. 2023). It was established for *Himalaeaunica* Hreblay & L. Ronkay, 1998, which was described based on a single adult male from Xizang (Tibet) Autonomous Region, China (Hreblay et al. 1998). The second species, *Himalaeabatanga* Saldaitis, Benedek & Volynkin, 2022, was also described from a single adult male but originated from the highlands of Sichuan Province, China (Saldaitis et al. 2022), about 850 km east of the type locality of *H.unica*. *Himalaeabatanga* differs from *H.unica* in having shorter antennal pectination, longer forewings, and different male genitalia, but the decision to consider it a separate subgenus was left until more comparative material was available. Recently, two adult males resembling *H.batanga* were collected in Xizang, about 530 km west of the *H.batanga* type locality, and based on differences in male genitalia they are considered a new species, described below. This new species, together with *H.batanga*, forms a group distinct from *H.unica* in both external appearance and male genitalia and is therefore considered to belong to a new subgenus, which is also established and described below.

## ﻿Material and methods

### ﻿Material

Adult moths deposited in the following collections were examined, photographed and dissected: **AFM** – research collection of Alessandro Floriani (Milan, Italy); **HNHM** – Hungarian Natural History Museum (Budapest, Hungary); **XU** – Xizang University (Lhasa, China). Labels of adults are cited verbatim in quotation marks (“”), with lines separated by a slash (/).

### ﻿Abbreviations (apart from the depositories) used

**GS** – genitalia slide; **HT** – holotype; **PT** – paratype.

### ﻿Photography and postprocessing

Adults were photographed using an Olympus C7070CW, a Canon EOS 5D mark II with a Canon EF 100mm f/2.8L Macro IS USM lens, and a Canon EOS 5D Mark VI. Slides were photographed using the LEICA EZ4 W. All images were processed with Affinity Photo 2 and Affinity Publisher 2.

### ﻿Genitalia dissection

Dissection was done following Hardwick (1950). The phallus was extracted and vesica was everted (Mikkola 2007; Zlatkov et al. 2022). The clasping apparatus and phallus were stained with Evans blue (Evans and Schulemann 1914; Cooksey 2013). The dissected genitalia were rinsed in 50%, 70%, and 96% ethanol and mounted on a microscope slide in Euparal (Gilson 1906; Neuhaus et al. 2017) and covered with a cover slip. Slides were deposited in the same collections as the dissected adults.

### ﻿Note on description and diagnosis paragraphs

Wing pattern nomenclature follows Forbes (1954); genitalial nomenclature follows Volynkin (2024).

## ﻿Taxonomic part

### ﻿ Himalaea

Taxon classificationAnimaliaLepidopteraNoctuidae

Hreblay & L. Ronkay, 1998

F303C7ED-B942-5A38-BA86-BE3FFF037E10

Himalaea Hreblay & L. Ronkay in Hreblay et al. 1998: 135. Type species: Himalaeaunica Hreblay & L. Ronkay, 1998, by original designation.

#### Diagnosis.

Adult males have bipectinate antennae and brownish-grey coloration. The main diagnostic feature in the clasping apparatus is an enlarged outgrown ampulla (digitus sensu Saldaitis et al. 2022), whereas the ampulla of other Psaphidini, if present, is smaller and does not rise above the surface of the valva. The vesica has anterior and posterior elasmas at the base of the vesica ejaculatorius, which are flat, sclerotized plates or raised, volumetric, sclerotized plates with serrated surfaces. Elasma occur in some other genera of Psaphidini: *Valeria* Stephens, 1829, *Meganephria* Hübner, 1820, *Benedekia* Ronkay, Ronkay, Gyulai & Hacker, 2010, and *Allophyes* Tams, 1942; however, the combination of robust ampulla and both anterior and posterior elasmas are unique within the tribe.

##### ﻿Taxonomic synopsis

**Subgenus Himalaea Hreblay & L. Ronkay, 1998**

**Himalaea (Himalaea) unica Hreblay & L. Ronkay, 1998**

**Subgenus Macrohimalaea subgen. nov.**

**Himalaea (Macrohimalaea) batanga Saldaitis, Benedek & Volynkin, 2022, stat. nov.**

**Himalaea (Macrohimalaea) silvana sp. nov.**

### ﻿ Macrohimalaea

Taxon classificationAnimaliaLepidopteraNoctuidae

Chen, Prozorov & Saldaitis subgen. nov.

E971FFF7-B47C-510F-BB38-B4095DF9ABA9

https://zoobank.org/4089696B-9450-49FC-8EBA-B2D2D3444167

Figs 2–4 , 6 , 7–8

#### Type species.

Himalaea (Macrohimalaea) silvana sp. nov., here designated.

#### Diagnosis.

**Figures 1–4.**
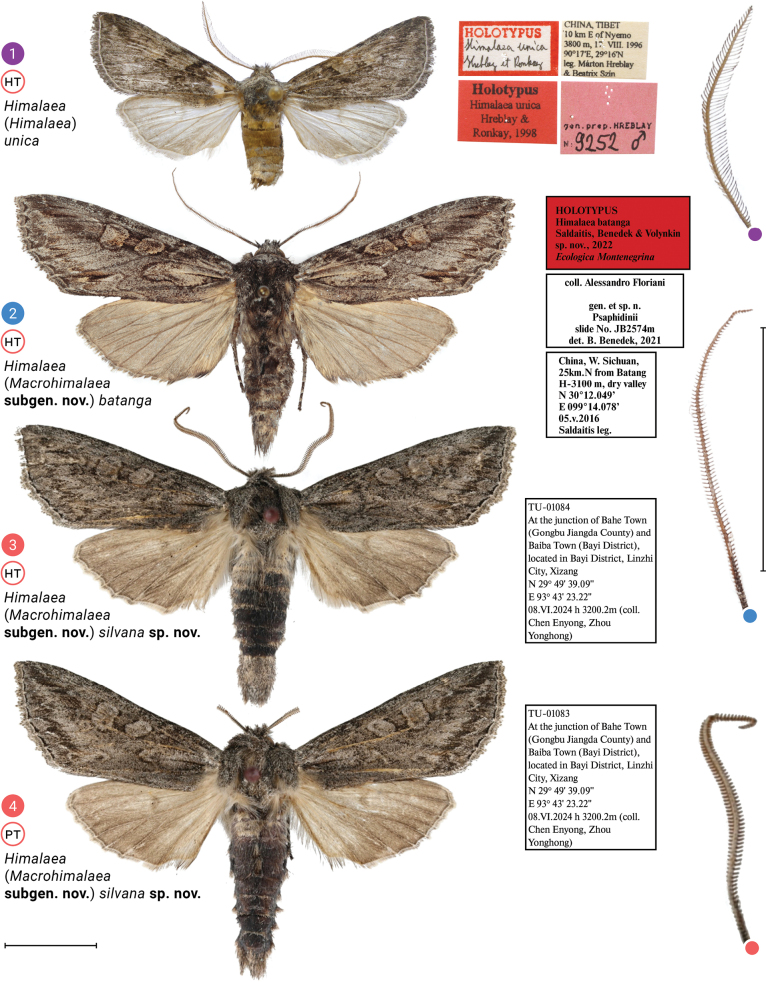
Adult males and enlarged antennae of *Himalaea* spp.; depositories of adults. **1.**HNHM (photo © B. Tóth); **2.**AFM; **3, 4.** TU. Scale bars: 1 cm.

**Figures 5, 6.**
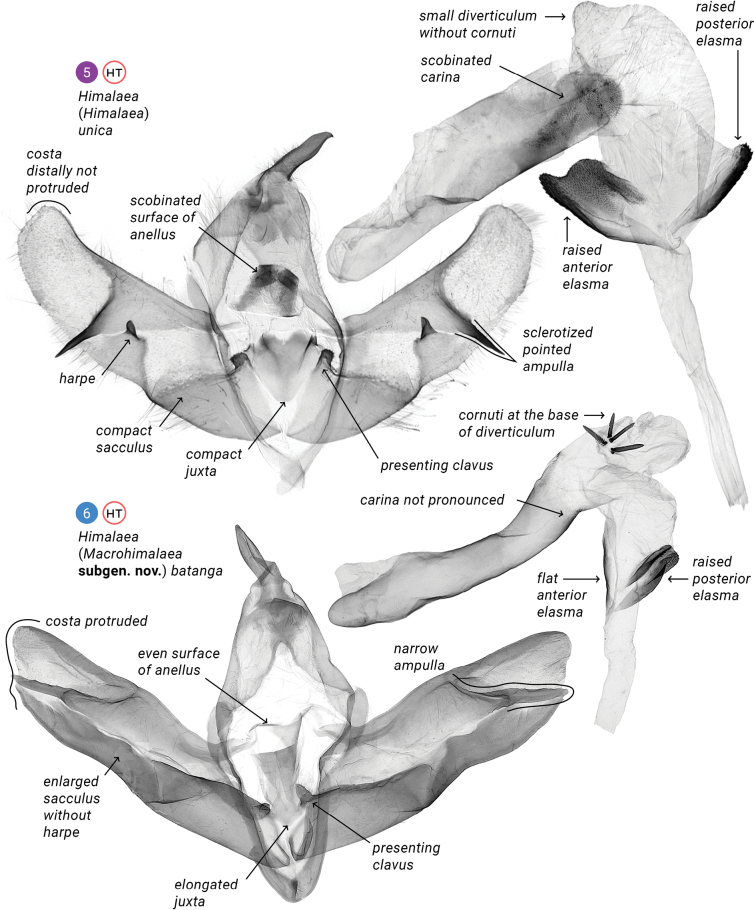
Male genitalia of *Himalaea* spp.; depositories of slides. **5.**HNHM (photo © B. Tóth); **6.**AFM.

**Figures 7, 8.**
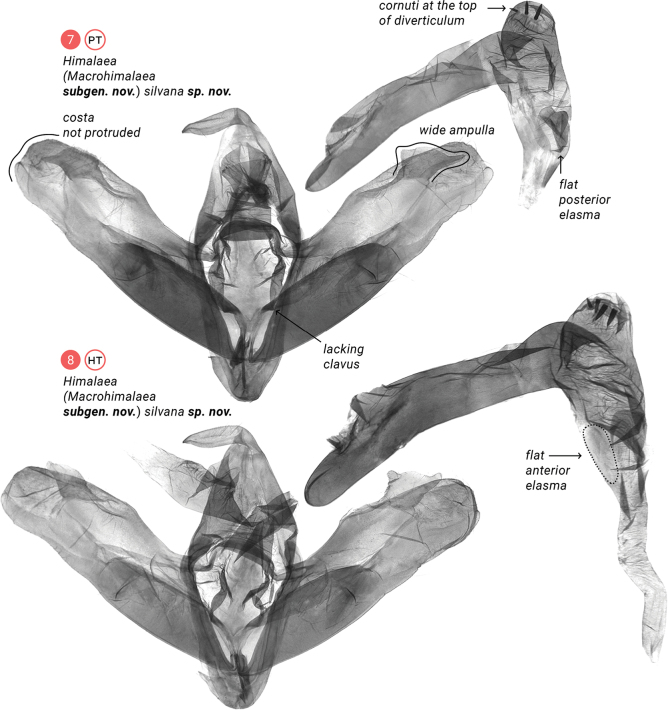
Male genitalia; depository of slides: XU.

The new subgenus differs from the nominotypical one in having shorter rami, and 1.3–1.5 times longer forewings with tapered apices (compare Fig. 1 with Figs 2–4). The male genitalia have an even-surfaced anellus, a dorsad elongated juxta, a sclerotized costa reaching the apex of the cucullus, a less-sclerotized ampulla lacking a harpe, an unpronounced carina, larger dorsal diverticulum bearing cornuti, and flat anterior elasma (compare Fig. 5 with Figs 6–8).

#### Description.

**Adult male** (Figs 2–4). ***Antenna*** bipectinate, ***rami*** gradually elongating towards mid third of antenna and then gradually shortening in distal third. ***Head***, ***thorax***, and ***abdomen*** dorsally brown-mottled, abdomen dark brown to blackish brown. ***Forewing*** 23.3 mm long; somewhat triangular, elongate and relatively narrow with obtuse tornal angle, slightly crenulate outer margin, prominence at M3, and tapered apex. Pattern consists of basal, medial, subterminal and terminal fields, with subterminal field being the lightest; dark basal streak within claviform spot bordered with dark wavy antemedial line; orbicular spot a little smaller than reniform spot, both with dark contour; dark, crenulate postmedial line; blurred, dark streak from posterior margin of reniform spot towards apex of wing; blurred, dark, zigzag subterminal line; more or less pronounced dark tornal streak; and dark terminal line split interrupted at veins. Cilia brown-mottled. ***Hindwing*** somewhat triangular with rounded apex and slightly crenulated outer margin; light-colored, getting darker at distal margin of discal cell. Cilia brown-mottled. **Male genitalia** (Figs 6–8). ***Uncus*** somewhat digitiform, basally bent at around 90°, apically narrowed to pointed apex. ***Tegumen*** a band narrowing towards connection with vinculum. ***Vinculum*** somewhat longer than tegumen, ventrally forming U-shaped saccus. ***Valva*** elongate, about the size of tegumen-vinculum complex, with well-pronounced sclerotized costa and sacculus, membrane between them, and membranous valvula, all sparsely covered with setae; distal third of costa less sclerotized, medially connected with relatively short (in comparison to ***Himalaea***) editum bearing ampulla; sacculus narrowing towards barely protruded rounded distal apex; both valvae joint with tendon. ***Juxta*** an elongated plate narrowing distally. ***Anellus*** small with smooth surface. ***Phallus*** as long as valva, slightly bent medially, with elongate coecum and wide entrance of ductus ejaculatorius; ***vesica*** somewhat conical, downcurved, with dorsal spherical diverticulum bearing three or four cornuti; at base of vesica ejaculatorius, anterior elasma flat, posterior elasma may be raised or flat. **Adult female** unknown.

#### Etymology.

The name *Macrohimalaea* is a derivative from *Himalaea*, the type subgenus of the genus, and prefix *macro*- for big or large to indicate that the specimens of *Macrohimalaea* are larger than the ones of *Himalaea*.

### ﻿ Himalaea (Macrohimalaea) silvana

Taxon classificationAnimaliaLepidopteraNoctuidae

Chen, Prozorov & Saldaitis sp. nov.

2C62882A-6B64-5190-ADBA-B24E669AC2CE

https://zoobank.org/D214E41C-96A9-4AA7-AAC9-5BA0270C2096

Figs 3 , 4 , 7 , 8

#### Type.

***Holotype***: • 1 male, “TU-01084/ At the junction of Bahe Town (Gongbu Jiangda County) and Baiba Town (Bayi District) / Baiba Town / Bayi District, Linzhi City, / Xizang / N 29° 52’ 22.09” / E 92° 31’ 31.96” / 23.8.2004 h 4203.7m (coll. XU),” GS prepared by Chen Enyong (XU).

***Paratype***: • 1 male, same data but “TU-01083” (XU).

#### Diagnosis.

The new species differs from H. (M.) batanga in having thicker rami, less contrasting forewings, and slightly darker hindwings (compare Figs 3, 4 with Fig. 2). The male genitalia which are lacking a clavus have wider ampulla, an unprotruded costa, a downcurved phallus, cornuti at the top of the dorsal diverticulum, and flat posterior elasma (compare Figs 3, 4 with Fig. 2). The new species was collected within the Northeast Himalayan subalpine coniferous forest ecoregion, which contrasts with H. (M.) batanga, which was collected within the Southeast Tibet shrublands and meadows.

#### Description.

**Male** (Figs 3, 4). ***Antenna*** bipectinate, ***rami*** gradually elongating towards mid third of antenna and then gradually shortening in distal third. ***Flagellum*** covered with greyish scales. ***Head*** dorsally, ***patagium*** and ***tegula*** grey-mottled. ***Thorax*** dorsally dark grey-mottled. ***Abdomen*** dorsally greyish-brown mottled at tergites II to III and at distal tip, whereas tergites IV to VIII covered with blackish scales. ***Forewing*** 23.3 mm long; somewhat triangular, elongate and relatively narrow with obtuse tornal angle, slightly crenulate outer margin, prominence at M3, and tapered apex. Pattern consist of greyish-brown mottled basal, medial, subterminal and terminal fields, with subterminal field being the lightest; dark-brown, narrow, basal streak inside light-greyish-brown claviform spot bordered with dark-brown, wavy antemedial line; orbicular and reniform spots with light-brown and sparse dark-brown scales and dark-brown contour; dark-brown, crenulate postmedial line; blurred, dark-brown streak from posterior margin of reniform spot towards apex of wing; blurred, dark-brown, zigzag subterminal line with whitish posterior streaks; more or less pronounced dark-brown tornal streak; and blackish terminal line interrupted at veins. Cilia greyish-brown mottled. ***Hindwing*** somewhat triangular with rounded apex and slightly crenulated outer margin. Light brown, getting darker at distal margin of discal cell and towards outer margin. Cilia brown-mottled. ***Male genitalia*** (Figs 7, 8). ***Uncus*** somewhat digitiform, basally bent at around 90°, apically narrowed to pointed apex. ***Tegumen*** a band narrowing towards connection with vinculum. ***Vinculum*** somewhat longer than tegumen, ventrally forms U-shaped saccus. ***Valva*** elongate, about the size of tegumen–vinculum complex, with well-pronounced sclerotized costa and sacculus, membrane between them, and membranous valvula, all sparsely covered with setae; distal third of costa less sclerotized, medially connected with rather short editum bearing somewhat trapezoidal ampulla with wavy distal margin and two lateral rounded apices, posterior one two times longer than anterior one; sacculus narrowing towards barely protruded rounded distal apex; both valvae joint with tendon. ***Juxta*** an elongated, distally narrowing plate. ***Anellus*** small, with smooth surface. ***Phallus*** as long as valva, slightly downcurved medially, with elongate coecum and wide entrance of ductus ejaculatorius; ***vesica*** somewhat conical, downcurved, with dorsal spherical diverticulum distally bearing three or four aligned cornuti, two elasmas pronounced at base of vesica ejaculatorius: anterior one somewhat oval, with fuzzy margin, weakly sclerotized (Fig. 8), and posterior one somewhat oval or heart-shaped with well-pronounced proximal margin and fuzzy distal margin, sclerotized harder than anterior one. **Female** unknown.

#### Biology and distribution (Figs 9–11).

The type series was collected from an altitude of 3,200 m on 23 August between 21:10 p.m. and 1:20 a.m. when the temperature was 15–16 °C and the relative humidity was around 60%. Adult moths collected there belonged to the families Noctuidae, Geometridae, Lasiocampidae, and Cossidae, with Noctuidae dominating in both numbers of species and individuals. The collection site was at the bottom of an alpine valley where the plant community was primarily dominated by *Pinusdensata* and *Quercusaquifolioides*, while the main plant community at the valley floor consisted of small shrubs. The collecting site lies within the Northeast Himalayan subalpine coniferous forest ecoregion.

**Figures 9–11.**
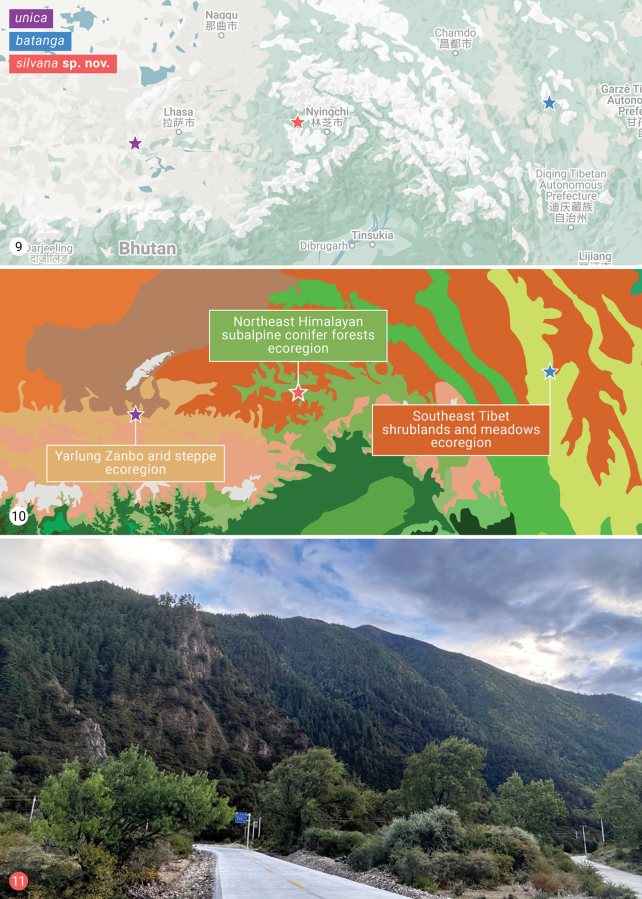
Collecting localities of *Himalaea* (Macrohimalaea subgen. nov.) *silvana* sp. nov. **9.** Physical map (map data ©2025 Google); **10.** Ecoregional map (image ©2025 TerraMetrics); **11.** Habitat.

#### Etymology.

The name *silvana* is a feminine derivative from the Latin noun *silva* meaning forest, given to the species for its occurrence near the Northeast Himalayan subalpine coniferous forest.

## Supplementary Material

XML Treatment for Himalaea

XML Treatment for Macrohimalaea

XML Treatment for Himalaea (Macrohimalaea) silvana

## ﻿Additional information

### Use of AI

No use of AI was reported.

### Author ORCIDs

Enyong Chen https://orcid.org/0000-0001-7503-844X

Alexey M. Prozorov https://orcid.org/0000-0002-5668-0741

Aidas Saldaitis https://orcid.org/0000-0003-0999-3996

Roman V. Yakovlev https://orcid.org/0000-0001-9512-8709

Günter C. Müller https://orcid.org/0000-0002-7024-0179

Yonghong Zhou https://orcid.org/0000-0002-2663-1885

### Data availability

All of the data that support the findings of this study are available in the main text.
